# A Case of Postpartum Pulmonary Edema Induced by Oxytocin

**DOI:** 10.7759/cureus.19590

**Published:** 2021-11-15

**Authors:** May Zaw, William Lim, Amber Latif

**Affiliations:** 1 Internal Medicine, BronxCare Health System, New York City, USA; 2 Internal Medicine, Richmond University Medical Center, New York City, USA; 3 Internal Medicine, University of California Los Angeles, Los Angeles, USA

**Keywords:** labor augmentation, labor induction, postpartum dyspnea, oxytocin, postpartum pulmonary edema

## Abstract

Postpartum dyspnea can be due to many causes, such as pulmonary embolism, amniotic fluid embolism, peripartum cardiomyopathy, but less frequently due to acute pulmonary edema. The incidence of acute pulmonary edema during pregnancy and in the postpartum period has been estimated to be around 0.08%. About half of the cases are attributed to tocolytic therapy. Herein, we present a case of a young woman presenting with acute hypoxia after induction of labor with oxytocin and found to have acute pulmonary edema. This case aims to illustrate and add to a growing body of literature regarding oxytocin-induced acute pulmonary edema and highlights the importance of recognizing the rare complication of oxytocin and necessary interventions to avoid complications. Oxytocin-induced pulmonary edema is a relatively uncommon condition, but physicians should have a high index of suspicion to initiate timely intervention and to avoid fetal complications.

## Introduction

The incidence of acute pulmonary edema during pregnancy and in the postpartum period has been estimated to be around 0.08%. Nearly half of the cases were attributed to tocolytic therapy or cardiac disease and the rest were due to either preeclampsia or iatrogenic volume overload [1]. In a few instances, oxytocin has been reported to be a cause of acute pulmonary edema [2-5]. We herein report a case of a young woman presenting with acute hypoxia after induction of labor with oxytocin and found to have acute pulmonary edema.

## Case presentation

A 28-year-old G2P0 41-week pregnant female with no significant past medical history presented with labor and delivery after an uneventful pregnancy. She underwent induction of labor by oxytocin after she was found to have chorioamnionitis. After unsuccessful induction with the administration of 10 units of oxytocin in 1000 ml of normal saline at a rate of 6 mU/min for 14 hours, a cesarean section was performed due to chorioamnionitis and abnormal fetal heart rate. Upon transfer to the recovery room, the patient started complaining of dyspnea and developed acute hypoxemic respiratory failure with a saturation of 60% on room air requiring noninvasive positive-pressure ventilation (NIPPV).

**Figure 1:**
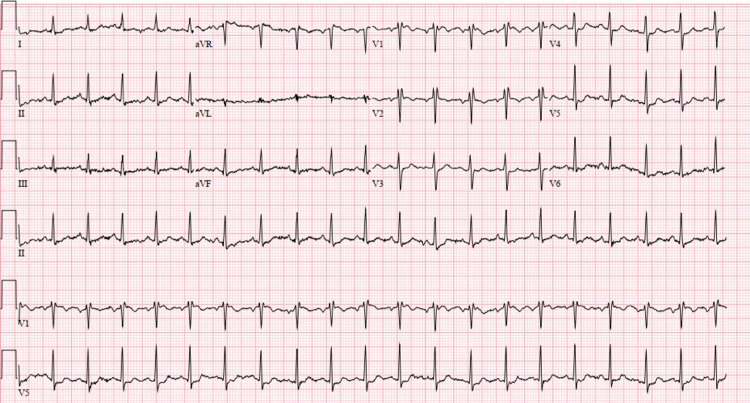
ECG showing sinus tachycardia with incomplete right bundle branch block, nonspecific T wave abnormality evident in the lateral leads.

Upon evaluation, the patient was alert and oriented but in respiratory distress with tachycardia and tachypnea, with reduced breath sounds bilaterally. The cardiac examination revealed a regular rate with normal first and second heart sounds with no murmur, rub, or gallop. The remainder of the abdominal, extremities, and neurological examinations revealed normal as well. Pertinent laboratory findings were pro-B-type natriuretic peptide of 2013 pg/mL with arterial blood gas studies showing pH of 7.302, pCO2 of 48.0 mmHg, pO2 66.3 mmHg, and bicarbonate 23.0 mmol/L. Transthoracic echocardiogram revealed mildly decreased left ventricular ejection fraction (LVEF: 50%) with grade 2 diastolic dysfunction and elevated pulmonary artery pressure. An ECG (Figure 1) showed sinus tachycardia with incomplete right bundle branch block, nonspecific T wave abnormality evident in the lateral leads.

Given the above clinical pictures, laboratory, and imaging findings, acute hypoxemic respiratory failure secondary to oxytocin-induced acute pulmonary edema was suspected and the patient was transferred to the ICU for close monitoring and the patient was started on IV diuretics. The patient was slowly tapered off from Bilevel Positive Airway Pressure (BiPAP) to non-rebreather (NRB) mask, then to nasal cannula (NC), and transferred out of the ICU.

**Figure 2:**
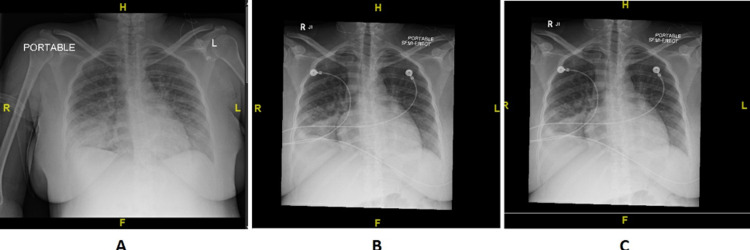
Chest X-ray showing improvement in vascular congestion.

Chest X-ray on initial presentation (Figure 2A), six hours after IV diuresis (Figure 2B), and six days (Figure 2C) after initial acute hypoxia showed marked improvement in vascular congestion.

## Discussion

Oxytocin is a neuropeptide hormone synthesized in the anterior pituitary and stored and released from the posterior pituitary. Oxytocin stimulates uterine contraction, cervical ripening, and dilation, and promotes clotting in the postpartum period [6]. Oxytocin is closely related to vasopressin in molecular structure and widely used for induction or augmentation of labor, prevention, and management of postpartum hemorrhage and is used as an adjunctive treatment of abortion. Common adverse effects include tachycardia, hypertension, nausea, vomiting, flushing, and headache [7]. Water intoxication and acute pulmonary edema are reported to be rare complications of oxytocin infusion. The risk of water intoxication and pulmonary edema from oxytocin is usually dose-related and occurs when it is administered in excessive doses with an electrolyte-free solution [8].

Oxytocin has an extensive effect on the cardiovascular system mediated by oxytocin receptors in both heart and blood vessels. Oxytocin has negative inotropic and chronotropic effects on the heart. It can also decrease vascular tone in arteries, resulting in lower blood pressure and a compensatory increase in heart rate. This, in turn, can lead to increased myocardial oxygen demand [9-11]. It can also increase water permeability by stimulating aquaporin-2 receptors in the collecting duct, leading to fluid retention [12].

Pulmonary edema can be divided into cardiogenic (due to high pulmonary capillary pressure) or noncardiogenic (due to factors other than elevated pulmonary capillary pressure). Oxytocin is postulated to induce both cardiogenic and noncardiogenic types of pulmonary edema due to its combined effect of decreased heart rate, contractility, and transient cardiac ischemia together with its anti-diuretic effect.

Peripartum cardiomyopathy is defined as idiopathic cardiomyopathy with ​​the development of heart failure toward the end of pregnancy or within five months following delivery, absence of another identifiable cause for the heart failure, and left ventricular systolic dysfunction with an LVEF of less than 45%. The mildly reduced ejection fraction (EF of 50%) in the presented case together with the temporal relationship between oxytocin infusion and symptom appearance as well as symptom resolution after therapy in this patient with no associated heart disease or cardiac risk factors indicated more towards the development of oxytocin-induced acute pulmonary edema than peripartum cardiomyopathy. Prompt recognition is essential in improving the prognosis of patients. Diagnosing can be challenging, requiring a combination of clinical evaluation, imaging, and laboratory investigations.

## Conclusions

We discussed a case of a young woman who developed signs of acute hypoxic respiratory failure from pulmonary edema in her immediate postpartum period. This case illustrates and adds to a growing body of literature regarding oxytocin-induced acute pulmonary edema and highlights the importance of recognizing the rare complication of oxytocin and necessary interventions to avoid complications. Oxytocin-induced pulmonary edema is a relatively uncommon condition, but proper caution needs to be exercised to initiate timely intervention and to avoid fetal complications.
